# Diversity and microevolution of CRISPR loci in *Helicobacter cinaedi*

**DOI:** 10.1371/journal.pone.0186241

**Published:** 2017-10-13

**Authors:** Junko Tomida, Yuji Morita, Keigo Shibayama, Ken Kikuchi, Tomohiro Sawa, Takaaki Akaike, Yoshiaki Kawamura

**Affiliations:** 1 Department of Microbiology, School of Pharmacy, Aichi Gakuin University, Nagoya, Japan; 2 Department of Bacteriology II, National Institute of Infectious Diseases, Tokyo, Japan; 3 Department of Infectious Diseases, Tokyo Women’s Medical University, Tokyo, Japan; 4 Department of Microbiology, Graduate School of Medical Sciences, Kumamoto University, Kumamoto, Japan; 5 Department of Environmental Health Sciences and Molecular Toxicology, Tohoku University Graduate School of Medicine, Sendai, Japan; Albert-Ludwigs-Universitat Freiburg, GERMANY

## Abstract

*Helicobacter cinaedi* is associated with nosocomial infections. The CRISPR-Cas system provides adaptive immunity against foreign genetic elements. We investigated the CRISPR-Cas system in *H*. *cinaedi* to assess the potential of the CRISPR-based microevolution of *H*. *cinaedi* strains. A genotyping method based on CRISPR spacer organization was carried out using 42 *H*. *cinaedi* strains. The results of sequence analysis showed that the *H*. *cinaedi* strains used in this study had two CRISPR loci (CRISPR1 and CRISPR2). The lengths of the consensus direct repeat sequences in CRISPR1 and CRISPR2 were both 36 bp-long, and 224 spacers were found in the 42 *H*. *cinaedi* strains. Analysis of the organization and sequence similarity of the spacers of the *H*. *cinaedi* strains showed that CRISPR arrays could be divided into 7 different genotypes. Each genotype had a different ancestral spacer, and spacer acquisition/deletion events occurred while isolates were spreading. Spacer polymorphisms of conserved arrays across the strains were instrumental for differentiating closely-related strains collected from the same hospital. MLST had little variability, while the CRISPR sequences showed remarkable diversity. Our data revealed the structural features of *H*. *cinaedi* CRISPR loci for the first time. CRISPR sequences constitute a valuable basis for genotyping, provide insights into the divergence and relatedness between closely-related strains, and reflect the microevolutionary process of *H*. *cinaedi*.

## Introduction

*Helicobacter cinaedi* is a gram-negative, motile, spiral, and microaerophilic bacterium, belonging to the family *Helicobacteriaceae*. It was first isolated in rectal swabs obtained from homosexual men in the 1980s [1]. Since 2000, the number of reports of *H*. *cinaedi* infections have been increasing. Examples of the diverse range of infections caused by *H*. *cinaedi* include proctocolitis, gastroenteritis, neonatal meningitis, localized pain, rash, and bacteremia [2]. This organism is difficult to culture and therefore difficult to isolate compared with other *Helicobacter* spp. and as a result its biological and clinical characteristics are less well understood [3]. In recent years, the risk of hospital (i.e., nocosomial) infections have become a problem, and this microorganism is considered a causative agent [4]. Furthermore, 30–60% of patients have recurrent symptoms; thus, *H*. *cinaedi* infections require careful handling in medical situations [5, 6, 7, 8]. Intriguingly, a potential association of *H*. *cineadi* infections with atherosclerosis is reportedly shown recently [3, 9, 10]. It is therefore important to assess the genetic diversity of epidemic strains to gain an understanding of the genetic distribution of the causative agents.

Clustered regularly interspaced short palindromic repeat (CRISPR) arrays and CRISPR-associated (Cas) proteins form the CRISPR-Cas system. The CRISPR array is composed of direct repeat sequences (repeats) and spacer sequences (spacers) that are derived from phages, plasmids, or other mobile genetic elements [11, 12]. This system provides adaptive immunity for many bacteria and most archaea [13, 14, 15]. The immune functions of the systems are carried out by Cas proteins, and the immunological mechanism involves a fragment of exogenous genetic elements becoming integrated into the CRISPR array, which generates a new repeat-spacer unit. Spacers appear to be integrated at one end (the leader end) of the CRISPR locus [13]. Thus, positional information represents a timeline of spacer acquisition events [14, 16, 17]. This can provide a unique, hypervariable locus that can be used to genotype organisms and provide insights into their divergence and relatedness [18, 19, 20].

In recent years, CRISPR-Cas typing has been used as effective approach to predict ancestral genotypes and patterns of descent within groups in, for example, *Clostridium difficile*, *Yersinia pestis*, *Salmonella enterica*, *Pseudomonas aeruginosa*, and *Riemerella anatipestifer* strains [17, 21, 22, 23, 24]. The analysis of polymorphisms among a bacterial population allows the reconstruction of the evolutionary history within a single species.

The CRISPR-Cas system can be divided into two classes and six types [25]. Class 1 systems, with multi-subunit effector complexes are comprised of multiple Cas proteins, including type I, type III, and putative type IV. Class 2 systems, with a single Cas protein effector, encompass type II, V and VI. Each group harnesses specific molecular mechanisms, although these systems have common main functional modules and play a role in immunity [17, 25, 26].

**Fig 1:**
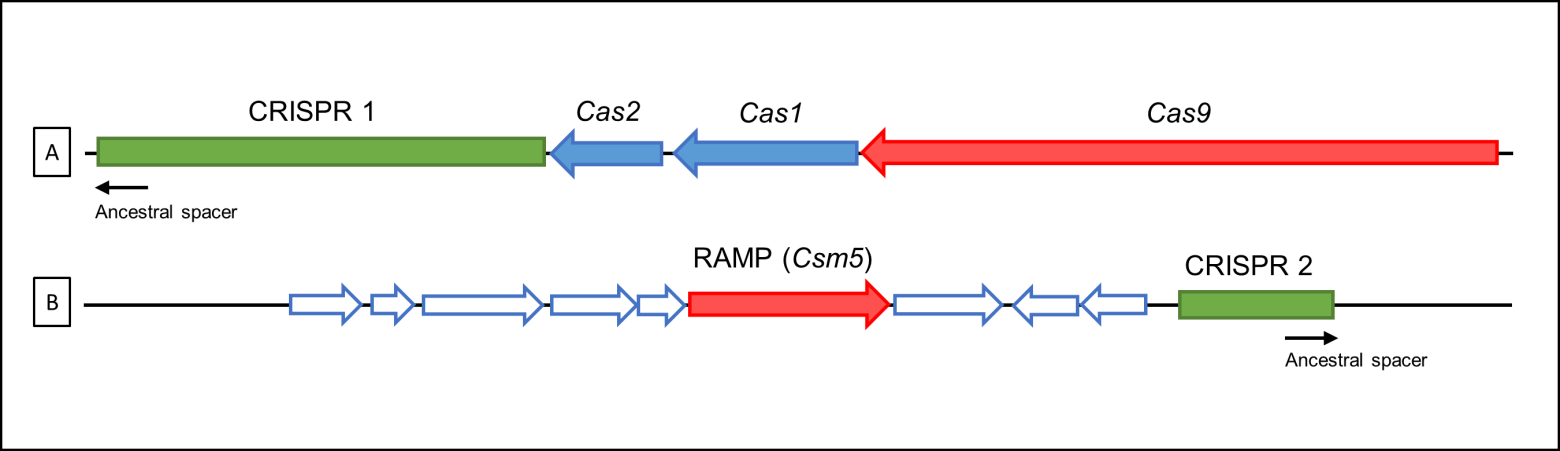
CRISPR-Cas locus architecture in *H*. *cinaedi* PAGU597^T^ strain. CRISPR loci in *H*. *cinaedi*: (A) CRISPR1, (B) CRISPR2. CRISPR1 and CRISPR2 loci were present in *H*. *cinaedi* genomes (AP012492) in 1362505–1364592 and 1889862–1890162, respectively. The signature gene for each type is shown in red (*cas*9 and RAMP for Type II and III, respectively). The universal *cas*1 and *cas*2 genes are blue. Accessory genes are white. CRISPR loci are shown in green. The arrows indicate the directions of the coding sequences.

The complete genome sequences of two *H*. *cinaedi* strains, CCUG18818^T^ (= ATCC BAA 847^T^ = PAGU 597^T^) and PAGU 611, have been reported [27, 28]. Moreover, while the presence of CRISPR-Cas loci in two *H*. *cinaedi* strains have been confirmed (Fig 1), the prevalence and diversity has not been explored in this species. The CRISPR-Cas system should provide useful information about strain characterization, lineage identification, and epidemiology.

Multilocus sequence typing (MLST) is a genotyping method based on the nucleotide sequences of seven housekeeping genes, which are used to assign different alleles to sequence types (ST) and clonal complexes. MLST has been widely used in molecular epidemiology and population biology in *Helicobacter* species [29, 30, 31], and has been proven useful for typing other *H*. *cinaedi* strains [32].

Genotyping analysis is crucial in terms of understanding the epidemiology of transmission; thus, the aim of the present work was to systematically investigate the prevalence and diversity of CRISPR loci in *H*. *cinaedi*. In this study, we developed a CRISPR sequence analysis for *H*. *cinaedi* and compared the results with MLST analysis.

## Materials and methods

### *H*. *cinaedi* strains and growth conditions

*H*. *cinaedi* strains were isolated from clinical materials at 6 different hospitals throughout Japan between 2004 and 2014 (S1 Table). Strains were cultured on Tryptic soy broth (Difco Laboratories) with 5% defibrinated horse blood and 1.5% Agar (Wako Pure Chemical Industries), and incubated at 37°C for 3 days under microaerobic conditions (6% O_2_, 7% CO_2_, 7% H_2_, 80% N_2_) generated by ANOXOMAT apparatus (MART II, MART Microbiology B.V.). Clinical isolates were identified as *H*. *cinaedi* by gram staining and a *H*. *cinaedi*-specific PCR approach [33]. In addition to the Japanese isolates, we assayed 6 reference strains isolated in other countries: *H*. *cinaedi* PAGU 597^T^ (= CCUG 18818^T^, isolated in the USA), PAGU 640 (= CCUG 19504, Canada), PAGU 1744 (= CCUG 19218, the USA), PAGU 1749 (= CCUG 38648, Sweden), PAGU 1752 (= CCUG 43522, Australia), and PAGU 1753 (= CCUG 44719, Sweden). The extraction of *H*. *cinaedi* genomic DNA was performed as reported previously [34]. The sequences of two complete genomes for *H*. *cinaedi* strains (PAGU597^T^, AP012492; PAGU611, AP012344) were obtained from the DNA Data Bank of Japan (DDBJ, http://www.ddbj.nig.ac.jp/index-j.html).

### CRISPR loci analysis

Primer pairs were designed to amplify the full CRISPR loci, respectively CRISPR1_Forward (5’-CAATTTAGAAAACGCAGAGCC-3’) and CRISPR1_Reverse (5’- GATATGATTTACCCTGCGGAAG-3’), and CRISPR2_Forward (5’- TGTCATACTGAGACTTTTGCC-3’) and CRISPR2_Reverse (5’- GCTACCCAAAGTCGCCAAAAC-3’). Other primers used for sequencing are listed in S2 Table. Amplification parameters consisted of 35 cycles of denaturation at 94°C for 15 s, annealing at 55°C for 15 s, and extension at 72°C for 2 min. PCR products were sequenced using PCR primers and sequencing primers, designed based on the spacer sequences. Sequence assembly and editing were performed with the DNASIS Pro Version 3.02 (Hitachi Solutions) and MEGA 6. The information pertaining to the CRISPR locus including position, length, and content were acquired from the CRISPR web server (http://crispr.i2bc.paris-saclay.fr/) [35]. Clustal X software was used to investigate the homology of the sequences of the CRISPR region possessed by each strain. The aligned sequences were compared by detecting identical spacers.

Visual representation of the CRISPR arrays was performed as previously described [21, 36]. The repeat sequences were removed for each array and the list of spacers was focused on the ancestral spacer on the left-hand side. Each spacer within the array was visually represented by a box. This allowed a comparison of conserved arrays by aligning spacers from the ancestral end. Spacer genotyping was based on common ancestral spacers. A matrix of zeros and ones was calculated, depending on the presence or absence of spacer arrays for every strain. The dendrogram was derived from the matrix of correlation distances by using the Jaccard similarity coefficient with the Dendro-UPGMA program (UPGMA), with a dendrogram construction utility (DendroUPGMA, http://genomes.urv.cat/UPGMA/index.php) [37]. CRISPRTarget (http://bioanalysis.otago.ac.nz/CRISPRTarget/crispr_analysis.html) [38] was utilized to predict the presence of possible protospacers. All spacer sequences were used for homology searching to find potential protospacers with >90% sequence identity [21].

### Multilocus sequence typing

Primers and PCR conditions for the seven housekeeping genes were as described in a previous report [32]. After confirming the single amplification products on 1% agarose gels, sequences were determined using a BigDye Terminator v3.1 Cycle Sequencing Kit (Applied Biosystems) and an automatic DNA sequencer (3130 Genetic Analyzer, Applied Biosystems). Allelic MLST sequences were analyzed using the PubMLST website (http://pubmlst.org/). Different STs and CCs were assigned using the *H. cinaedi* MLST database (http://pubmlst.org/hcinaedi/). The phylogeny for the 42 isolates was estimated by concatenated sequences using the neighbor-joining method [39]. Clustal X software was used to align the sequences [40], and calculate the genetic distances. The dendrogram was constructed using NJplot program [41] and MEGA 6 [42].

## Results

### The CRISPR loci structure in *H*. *cinaedi*

Based on genomic analysis [27, 28], CRISPR loci are flanked by *cas* genes encoding Cas proteins (Fig 1). Three *cas* genes (*cas2*, *cas1* and *cas9*, in this order) were located upstream of the CRISPR1, which is consistent with a type-II system [43]. Cas1 and Cas2 are the core proteins of the CRISPR-Cas system [15]. Cas9 protein sequencing analysis is consistent with the classification of type II systems characterized to date [44]. To determine the type of *H. cinaedi* CRISPR-Cas system, we obtained Cas9 amino acid sequences from Gram-negative type II system-containing bacteria, as previously described [17], and compared them with the Cas9 sequences of *H. cinaedi* strains PAGU597^T^ and PAGU611. We constructed a multiple sequence alignment and phylogenetic tree for Cas9 (S1 Fig). The phylogenetic tree showed that the Cas9 sequences from two *H. cinaedi* were closely related to those of *Campylobacter jejuni* subsp. *jejuni* NCTC11168, and formed part of the subtype II-C subcluster.

RAMP gene was located downstream of the CRISPR2 locus. *Cas*1 and *cas*2 genes were not found in CRISPR2 and the predicted length of the ORF for RAMP was 1782 bp—RAMP is a signature gene of the type III system [15].

The two CRISPR loci, CRISPR1 and CRISPR2, were identified for all *H. cinaedi* strains by CRISPR PCR and sequencing analysis. An average of 32 spacers (ranging from 4 to 63) were identified in CRISPR1 loci, while CRISPR2 loci had 6 spacers (ranging from 2 to 10). It has been reported that CRISPR repeats are composed of exact repeat sequences ranging from 24 to 48 bases long [45]. These sequences have also been shown to contain palindromes. The 5’ terminal portion of a repeat is normally composed of the sequence GTTT (G) and the 3’ terminus contains GAAA (C/G) [17, 46]. Generally, repeats associated with the type II system are weakly palindromic, and typically 36 bp in length [43]. CRISPR1 and CRISPR2 in *H. cinaedi* strains retained a 36-bp long repeat sequence. The consensus direct repeats associated with CRISPR1 contained a conserved 5’- GTTTTAGTCCCTTCTTAAACTTCTATATGCTAGAAT-3’. A conserved 5’- GTTTTAGTGGGACCCGATTTAAGGGGATTTGTATCA -3’ was present in CRISPR2.

### Distribution and conservation of CRISPR spacer arrays

**Fig 2:**
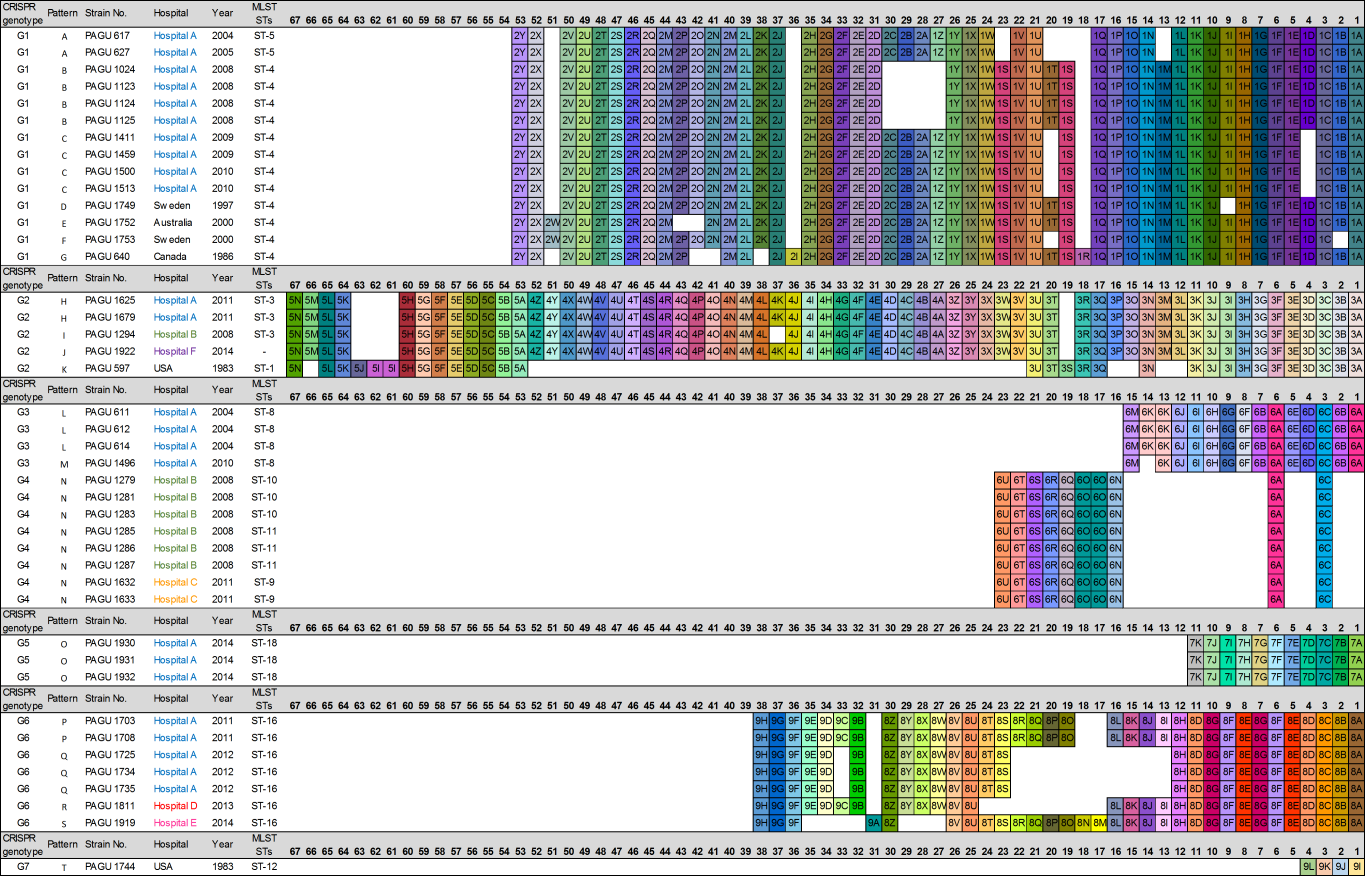
CRISPR spacer content and polymorphisms in CRISPR1. The CRISPR1 arrays from 42 *H*. *cinaedi* strains are represented graphically. The repeats have been eliminated and only spacers are shown. Identical spacers are shown as squares representing the same combination of numerals and letters, and aligned so that they have same number (apart from duplicate spacers). Spacer numbering is initiated at the ancestral end (right) towards the most recently acquired spacers per strain (light). Strains are listed by CRISPR genotype, CRISPR array pattern, strain number, hospital, year of isolation, and MLST sequence type.

**Fig 3:**
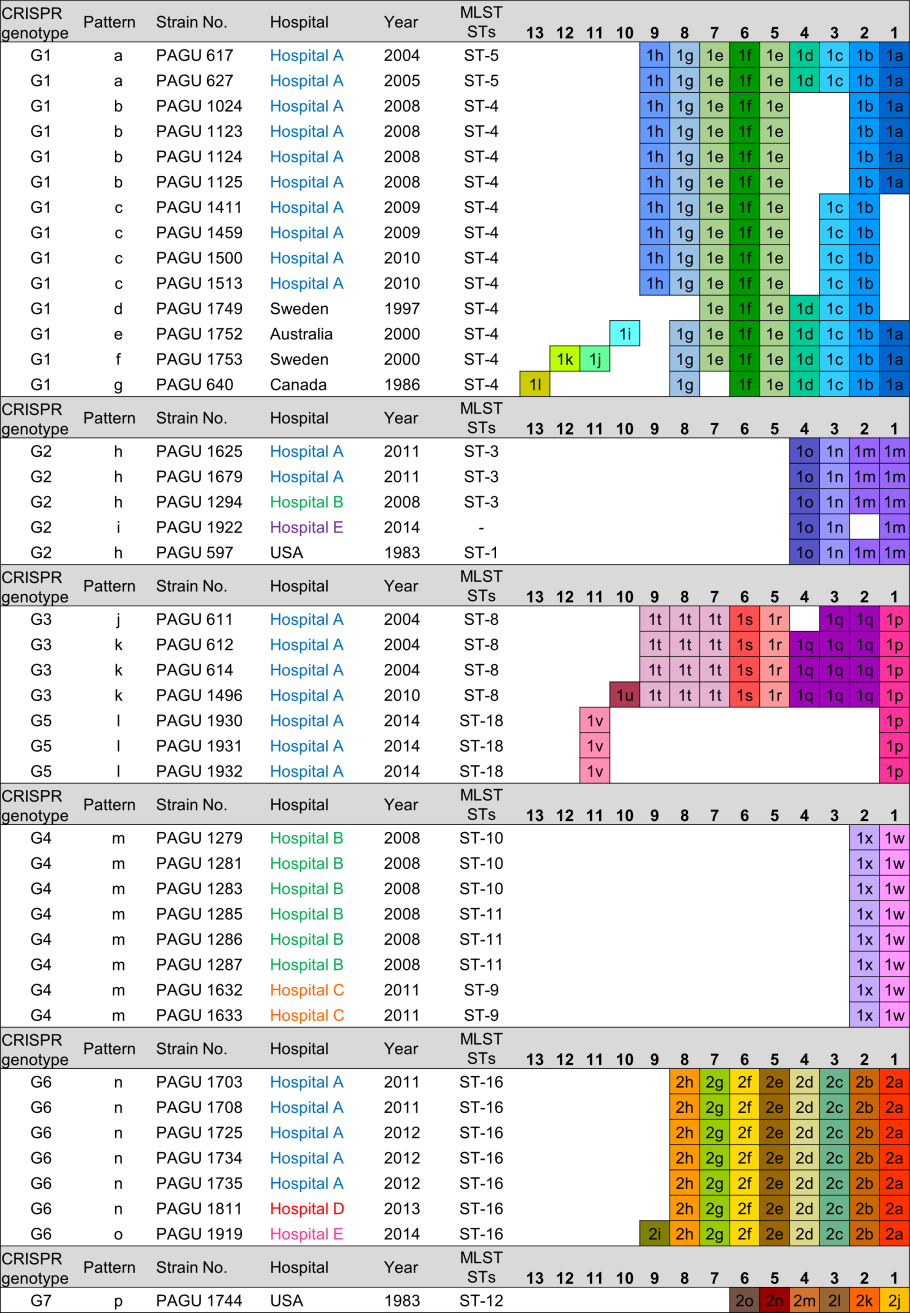
CRISPR spacer content and polymorphisms in CRISPR2. The CRISPR2 arrays from 42 *H. cinaedi* strains are represented graphically. Identical spacers are shown as squares representing the same combination of numerals and letters, and are aligned so that they have same number (apart from duplicate spacers). Spacer numbering is initiated at the ancestral (right) end towards the most recently acquired spacers per strain (left).

**Fig 4:**
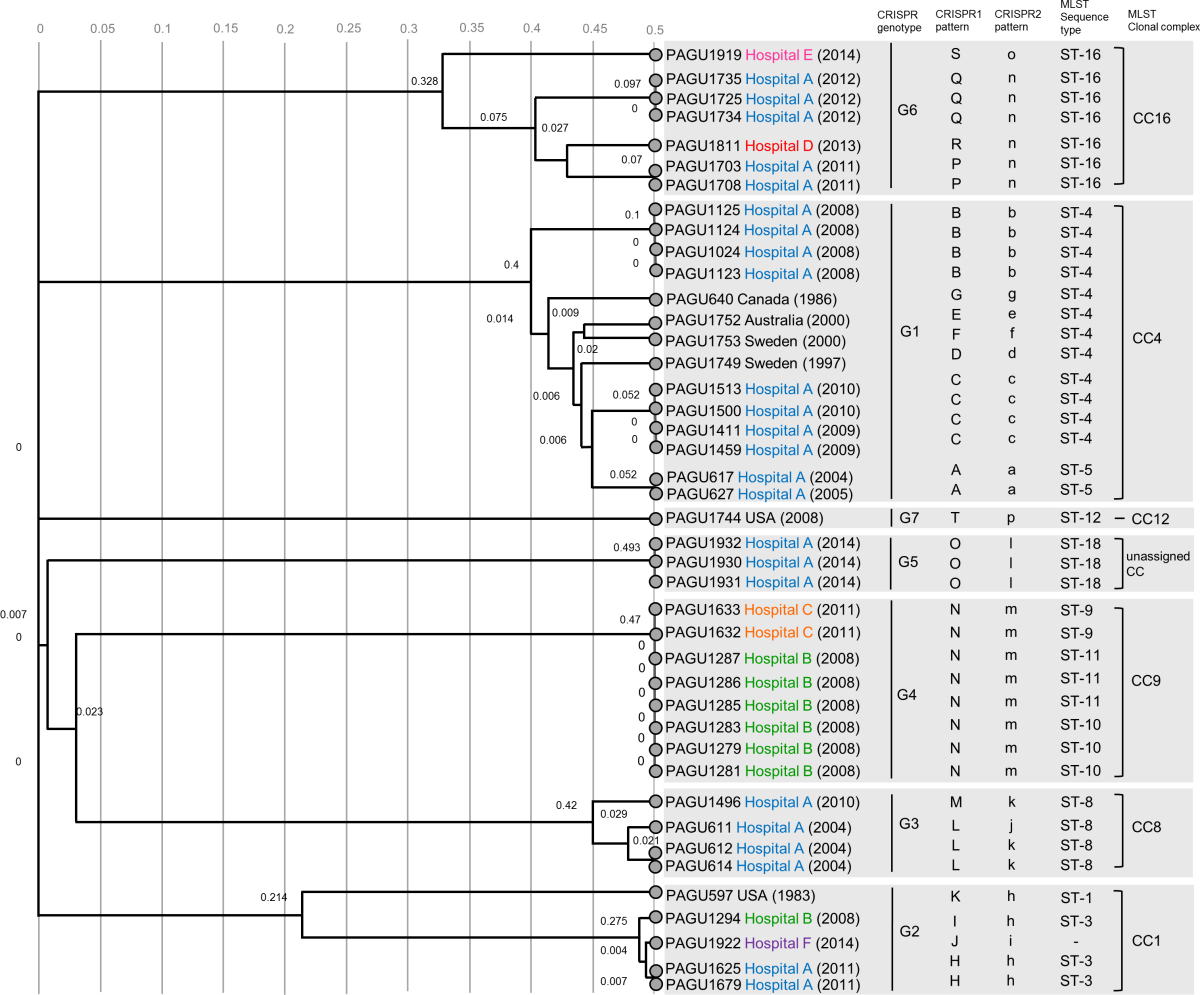
Unweighted Pair Group Method with Arithmetic Averages (UPGMA) dendrogram derived by comparing the spacer patterns for CRISPR1 and CRISPR2 profiles from 42 *H*. *cinaedi* strains. The scale indicates the genetic distances calculated by UPGMA method. All sequences are labeled by strain number, hospital, and year of isolation in parentheses. CRISPR genotypes, CRISPR1 and CRISPR2 patterns, MLST sequence type, and MLST clonal complexes are indicated. PAGU1922 strain was assigned to an unknown ST, indicated in quotes. PAGU1930, PAGU1931, and PAGU1932 were also not assigned to any clonal complexes. Strains assigned to ST-3, ST-4, ST-8, and ST-16, which had identical sequences at all seven loci in each ST, had different spacer distributions in CRISPR analysis. In some clinically relevant strains (PAGU 611, 1294, 1703, 1708 and 1811), the spacer distribution of CRISPR1 differed, even though that of CRISPR2 was same, and vice versa.

Identification of the spacer sequences from the CRISPR loci was conducted to evaluate the extent of genotypic diversity among the *H. cinaedi* isolates. We applied an approach to outline the distribution of conserved CRISPR arrays—identified by their ancestral spacer content—in all 42 strains. A conserved ancestral spacer implies commonality among the strains, whereas spacers acquired later may differ between related strains due to different exposures to foreign invasive DNA. The distribution of the identified ancestral spacers enabled the CRISPR arrays to be grouped by spacer organization. The spacer composition of CRISPR1 and CRISPR2 loci are indicated in Figs 2 and 3. We found 20 unique CRISPR1 patterns (CRISPR1 patterns A to T, Fig 2) and 16 unique CRISPR2 patterns (CRISPR2 patterns a to p, Fig 3). The 42 *H. cinaedi* strains were grouped into 7 different genotypes (G1—G7), according to the sequence spacer arrays (presence or absence) and ancestral spacers (Figs 2–4).

The six reference strains had different spacer arrays compared to the Japanese isolates. PAGU 640, 1749, 1752, and 1753 (from outside Japan) had the same ancestral spacer as genotype G1 and shared conserved spacers, in addition to unique spacers (spacer 1R, 2I, 2W of CRISPR 1; spacer 1i, 1j, 1k, 1l of CRISPR2). Unique spacers (spacer 3S, 5I, 5J of CRISPR1) were also present in PAGU 597^T^ isolated in the USA, which shared conserved spacers with genotype G2. In this study, the predecessor of genotype 2 had not been identified. It was predicted that a large spacer deletion occurred during expansion of the ancestral lineage, resulting in PAGU 597 strain. The spacer organization of PAGU 1744 from the USA was distinctive, and all spacers of the two loci were composed of unique nucleotide sequences.

### MLST typing

**Fig 5:**
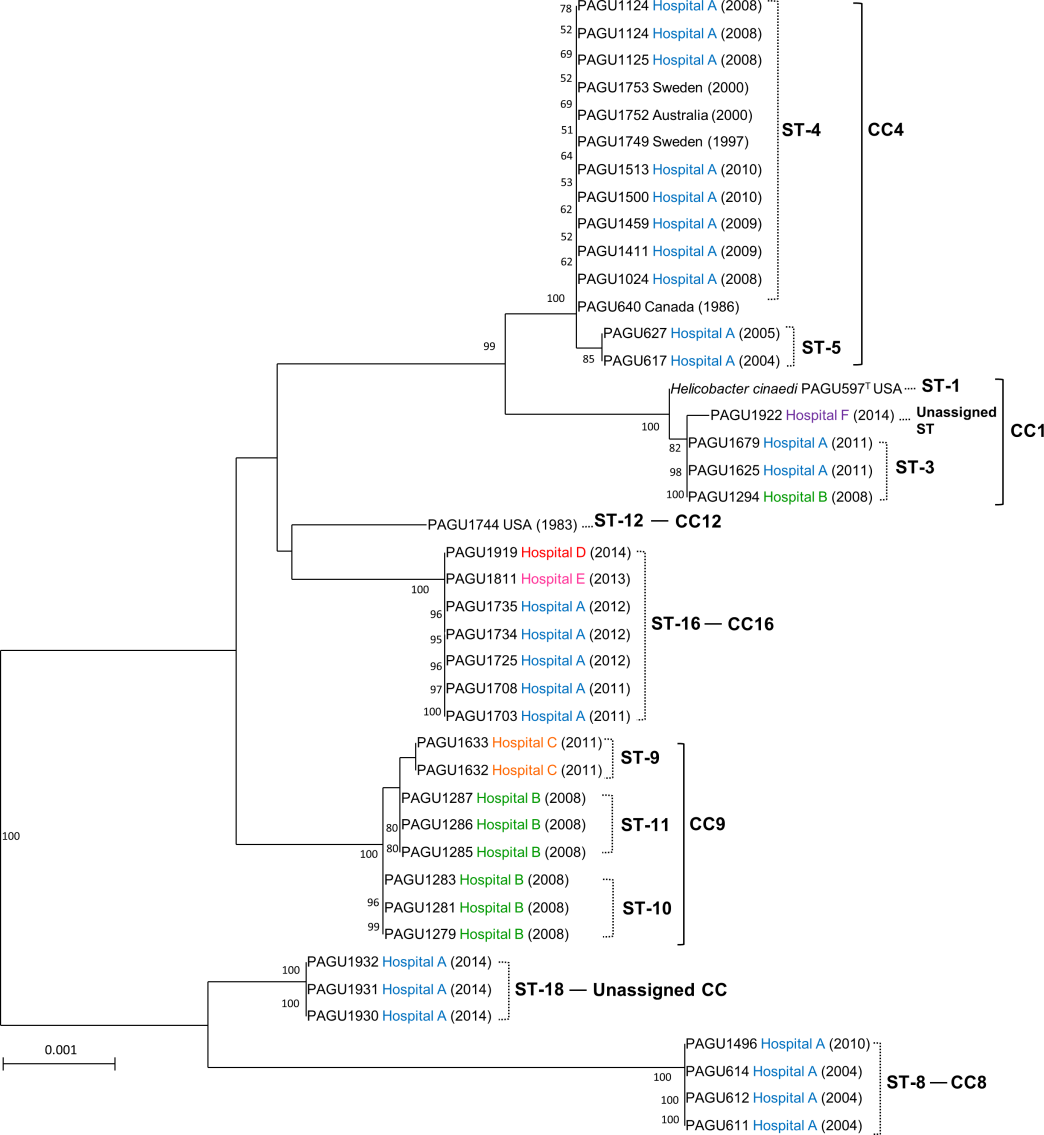
Phylogenetic tree of 11 STs of *H*. *cinaedi* isolates using MLST analysis. Phylogenetic analysis was generated with the neighbor-joining method. Numbers at the nodes represent bootstrap values > 50% (obtained from 100 resamplings). All sequences are labeled by strain number, hospital, and year of isolation in parentheses. The colors represent the different hospitals. Bars: 0.001 substitutions per nucleotide position.

A total of 11 different sequence types (STs) were identified among the 42 isolates (Table 1). PAGU 1922 had a different allelic profile and did not correspond with any ST belonging to CC1. Among the STs, 11 were assigned into 6 known CCs while ST-18 was unassigned. Based on the phylogenetic tree of MLST, the 36 clinical isolates from Japan were classified into 6 clusters, CC1 (4 isolates), CC4 (14 isolates), CC8 (4 isolates), CC9 (8 isolates), CC16 (7 isolates), and unassigned CC (ST18, 3 isolates) (Fig 5). These *H. cinaedi* isolates were collected from 5 hospitals in Japan, and the distributions within each hospital were compared. Hospital A obtained 24 isolates over 11 years, which were subsequently divided into 5 clusters (CCs 1, 4, 8, 16, and ST-18). The reference strains (PAGU 597^T^ and 1744) revealed slightly different sequences compared to the Japanese isolates, while PAGU 640, 1749, 1752, and 1753, which were classified as ST-4, had the same ST as the isolates from hospital A.

**Table 1:** MLST analysis of 42 *H*. *cinaedi* isolates.

Clonal complex	Sequence type	(No of isolates)	Allelic profile							CRISPR		
			23S	ppa	aspA	aroE	atpA	tktA	cdtB	Genotype	CRIRSPR1 pattern	CRIRSPR2 pattern
CC1	ST-1	(1)	1	1	1	1	1	1	1	G2	K	h
CC1	ST-3	(3)	1	1	1	1	1	4	1	G2	H, I	h
CC1	-	(1)	UA	1	1	1	1	4	1	G2	J	i
CC4	ST-4	(12)	3	3	3	1	2	2	1	G1	B, C, D, E, F, G	a, b, c, d, e, f, g
CC4	ST-5	(2)	3	3	3	1	4	2	1	G1	A	a
CC8	ST-8	(4)	3	4	4	3	2	3	2	G3	L, M	j, k
CC9	ST-9	(2)	3	2	2	2	2	1	2	G4	N	m
CC9	ST-10	(3)	4	2	2	2	2	1	2	G4	N	m
CC9	ST-11	(3)	2	2	2	2	2	1	2	G4	N	m
CC12	ST-12	(1)	5	5	2	5	5	1	3	G7	T	p
CC16	ST-16	(7)	4	3	2	2	6	4	3	G6	P, Q, R, S	n, o
UA	ST-18	(3)	6	3	6	3	3	1	3	G5	O	l

UA, unassigned

### Comparison of CRISPR analysis and MLST

Twelve MLST STs were identified among the 42 isolates, whereas there was a greater number of CRISPR patterns (20 CRISPR1 patterns, 16 CRISPR2 patterns, Table 1 and Fig 4), which indicated that CRISPR analysis has greater discriminatory power than MLST. Isolates assigned to ST-4 diversified into six distribution patterns (B, C, D, E, F and G) of CRISPR1 and six patterns of the CRISPR2 (b, c, d, e, f and g). Similarly, the strains assigned to ST-3, ST-8, and ST-16 differentiated into separate CRISPR1 patterns (ST-3, H and I; ST-8, L and M; ST16, P, Q, R, and S). Each sequence of seven housekeeping genes among the PAGU611 and PAGU1496 strains belonging to ST-8 demonstrated identical sequences at all seven loci. However, these strains were isolated at different times and, according to the distribution of CRISPR1 loci, it appeared that the spacer defect of PAGU1496 occurred between 2004 and 2010 in hospital A (spacer 6K, Fig 2). The diversity was revealed by determining the CRISPR sequences for strains assigned to the same ST in MLST analysis.

## Discussion

Reports on the number of *H. cinaedi* infections have been steadily growing, and the association of this bacterium with a variety of human infections and atherosclerotic diseases has received increasing attention in recent years [3, 10]. *H. cinaedi* is currently the most commonly reported enterohepatic Helicobacter isolated in humans. Kitamura *et al*. previously documented an outbreak of nosocomial *H. cinaedi* infections caused by direct person-to-person spread [6]. We have also received reports of a growing number of cases of nosocomial *H. cinaedi* infections in Japan. Indeed, this microorganism is recognized as a causative agent of nosocomial infections [4]. *H. cinaedi* strains were isolated from men and women of a broad age-range (from neonates to the elderly). Some patients had immunocompromised conditions, while others had not been in apparently immunocompetent [47]. *H. cinaedi* infections have been detected in hospitals throughout Japan, and we hypothesize that they are more common in Japanese hospitals than is currently recognized.

This study attempted to compare CRISPR arrays to gain an understanding of the diversity of *H. cinaedi*. The CRISPR1-*cas* locus possessed the minimum number of *cas* genes required to formulate the *cas* operon—a characteristic of subtype II-C [15]. The repeats of *H. cinaedi* CRISPR1 were 36 bp in length, which corresponded with type II systems. The *cas* components suggested that CRISPR2 of *H. cinaedi* strains resembles type III systems. *Cas*1 and *cas*2 genes were not found in the CRISPR2 loci, but in many organisms, the type III CRISPR–*cas* operons lack the *cas*1–*cas*2 gene pair [15].

Hospital A has been isolating *H*. *cinaedi* strains since 2004. Two genotypes of *H*. *cinaedi* (genotype G1 and G3) were found in 2004 in a comparison of the evolution of spacer organization over time. Genotypes G1 and G3 were distinguished by the presence of different ancestral spacer. Genotype G1 strains shared the ancestral spacers 1A and 1a. The ancestral spacers 6A and 1p were present in genotype G3. In a previous analysis using pulse field gel electrophoresis typing [6], the strains isolated from 2004 to 2005 in hospital A could be divided into two clusters (initial outbreak strain, subsequent outbreak strain). This clustering pattern was also supported by the phylogenetic tree of *hsp* gene, as well as the RAPD pattern. These findings are consistent with our results showing genotypes G1 and G3 by CRISPR analysis (Fig 4).

Our results not only provide information about the homology of the sequences in the CRISPR region, but also enable the process of spread to be traced via CRISPR arrays by showing the acquisition and deletion of spacers. Although genotype G1 strains have been circulating in hospital A since 2004, the arrangement of the spacers has frequently changed. These strains were subsequently isolated in the same hospital in 2008, 2009, and 2010.

Based on CRISPR distribution, genotype G1 isolates obtained in hospital A were further divided into three subtypes (genotype G1-I; PAGU 617 and 627, genotype G1-II; PAGU 1024, 1123, 1124, and 1125, genotype G1-III; PAGU 1411, 1459, 1500, and 1513). It could be considered that the predecessor of these subtypes was not identified in this study, and spacer deletions occurred while the genotype G1 isolates were spreading. These data show that CRISPR pattern can systematically distinguish closely-related strains, and reflect the microevolution of strains that are particularly relevant among the same genotypes.

Strains classified as genotype G3 were isolated for the first time in 2004 (PAGU611, PAGU612, and PAGU614), circulated without elimination for several years at the same hospital, and were again detected in patients in 2010 (PAGU1496). In addition to the two major genotypes G1 and G3, genotypes G2, G5, and G6 have also circulated since 2011 at hospital A.

Based on CRISPR analysis, all strains from hospital B were classified as genotype G4 except one (PAGU 1294). However, six of the isolated strains were grouped into two STs (ST-10 and ST-11) via MLST. The reason for dividing the strains into ST-10 and ST-11 was due to differences between two of the bases of the 23S rRNA sequence. Alignments of the sequences of the 23S rRNA gene showed that the nucleotides at positions 547659, 547760, and 548262 (the base order of the genomic sequence of *H*. *cinaedi* PAGU597^T^, AP012492) were G-T-T in ST-10 and G-C-C in ST-11, respectively. The nucleotide sequence of the above-mentioned site of the strain classified as ST-9 from hospital C is G-T-C. Thus, the distinction between the three STs classified as CC-9 is derived from only two base differences in the nucleotide sequence of 23S rRNA gene. In the 8 strains classified as CC-9, the nucleotide sequences of the other 6 genes were identical by MLST. A previous comparison of the 23S rRNA gene sequences has been reported for the strain isolated in hospital B, [48].These ST-10 and ST-11 strains were isolated from female and male patients, respectively, and it was reported that nosocomial infections could have occurred in these cases via the female or male toilets, respectively. Although the efficacy of sequencing analysis of the 23S rRNA gene has been described [49], the sequences of the 23 rRNA gene of the 4 strains assigned to ST-1 and ST-3 appeared identical, as did those of the 20 ST-4, ST-5, ST-8, and ST-9 strains in this study (Table 1). Therefore, the discrimination value of the 23S rRNA gene sequencing analysis of *H*. *cinaedi* strains is low.

The *gyrA* sequence is an appropriate marker with a high discrimination rate for the phylogenetic analysis of the *Helicobacter* genus [50]. We evaluated the genetic relationships of our isolates using *gyrA* and 16S rRNA gene sequences, which are the gold standard for phylogenetic analysis. The *gyrA* sequences of *H*. *cinaedi* isolates showed low diversity (S2 Fig), which led us to conclude that these sequences were useful for analysis within genus, but not within species. The 16S rRNA gene was further investigated for the analysis of *H*. *cinaedi* isolates within species (S3 Fig). It was generally thought that the 16S rRNA gene was insufficient for identification at the species level as a stand-alone technique in phylogenetic analysis, but the 16S rRNA phylogenetic tree yielded the same topology as MLST in *H*. *cinaedi* species. Thus, the phylogenetic analysis of the 16S rRNA gene was considered reliable for *H*. *cinaedi*, contrary to its use for other bacterial species.

In MLST analysis, the strains assigned to ST-3, ST-4, ST-8, and ST-16 had seven genes showing identical nucleotide sequences within each ST group, and no diversity was observed. Meanwhile, in CRISPR analysis, these strains had different spacer distributions, even within the same ST via MLST, and the strains belonging to one ST were divided into two or more CRISPR patterns (Table 1).

The 12 STs were divided into 20 CRISPR patterns, and CRISPR typing is considered to have higher discriminatory power than MLST. In addition, the spacer array of CRISPR does not only distinguish between strains, but also provides useful background information about the evolution of the strains. We can predict the relevance of isolates depending on whether they have a common ancestral spacer. For these reasons, CRISPR analysis is thought to be efficient and provide more information than other genotyping methods.

We have described the epidemiological analysis of *H*. *cinaedi* isolates using CRISPR arrays. The polymorphisms among the organization of spacers reflect the adaptation process of *H*. *cinaedi*. Thus, the distribution of CRISPR spacers may assist in the study of nosocomial *H*. *cinaedi* infections, and may be useful for typing *H*. *cinaedi* isolates and elucidating how they spread. CRISPR-Cas system data will contribute to a better understanding of the origins and microevolution of this microorganism.

## Supporting information

S1 Table Strains included in this study. (DOCX)

S2 Table Primers designed for the CRISPR sequence amplification of *Helicobacter cinaedi*. (DOCX)

S1 Fig Phylogenetic tree for Cas9 from Gram-negative type II system-containing bacteria. Cas9 proteins from Gram-negative type II system-containing bacteria are referenced [17]. A phylogenetic tree based on Cas9 proteins was constructed by the neighbor-joining method. *H*. *cinaedi* strains PAGU597^T^ and PAGU611 are shown in red. Two Cas9 protein sequences were obtained from the DDBJ (Accession Nos. AP012492 and AP012344, respectively).
(PDF)

S2 Fig Evolutionary relationships of *H*. *cinaedi* isolates based on the *gyrA* gene. Phylogenetic analysis of the *gyrA* gene was performed using the neighbor-joining method. All sequences are labeled by strain number, hospital, and year of isolation. The colors represent the different hospitals. Bars: 0.02 substitutions per nucleotide position.
(PDF)

S3 Fig Evolutionary relationships of *H*. *cinaedi* isolates based on the 16S rRNA gene. Phylogenetic analysis of the 16S rRNA gene was performed using the neighbor-joining method. Numbers at the nodes represent bootstrap values > 50% (obtained from 100 resamplings). All sequences are labeled by strain number, hospital, and year of isolation. The colors represent the different hospitals. Bars: 0.002 substitutions per nucleotide position.
(PDF)
