# Supplementary material for: Diversity and microevolution of CRISPR loci in Helicobacter cinaedi
Source: PLoS One. 2017 Oct 13;12(10):e0186241. doi: 10.1371/journal.pone.0186241 (PMC5640232; doi:10.1371/journal.pone.0186241)
Supplement: S1 Table — (DOCX) [file pone.0186241.s001.docx]

S1 Table. Strains included in this study.

| Species | Strain No. | Isolated from | year of isolation | Genbank Accession No. | | Sequence type | CRISPR1 Pattern | CRISPR2 Pattern |
| --- | --- | --- | --- | --- | --- | --- | --- | --- |
|  |  |  |  | CRISPR 1 | CRISPR 2 |  |  |  |
| *H. cinaedi* | PAGU 597 (=CCUG 18818^T^) | USA, Seattle | 1980-1983 | AP012492 | AP012492 | ST-1 | K | h |
| *H. cinaedi* | PAGU 640 (=CCUG 19504) | Canada, Ottawa | 1986 | LC216939 | LC216979 | ST-4 | G | g |
| *H. cinaedi* | PAGU 1744 (=CCUG 19218) | USA, Seattle | 1980-1983 | LC216965 | LC217005 | ST-12 | T | p |
| *H. cinaedi* | PAGU 1749 (=CCUG 38648) | Sweden, Uppsala | 1997 | LC216966 | LC217006 | ST-4 | D | d |
| *H. cinaedi* | PAGU 1752 (=CCUG 43522) | Australia | 2000 | LC216967 | LC217007 | ST-4 | E | e |
| *H. cinaedi* | PAGU 1753 (=CCUG 44719) | Sweden, Karlskrona | 2000 | LC216968 | LC217008 | ST-4 | F | f |
| *H. cinaedi* | PAGU 611 | Hospital A, Japan | 2004 | AP012344 | AP012344 | ST-8 | L | j |
| *H. cinaedi* | PAGU 612 | Hospital A, Japan | 2004 | LC216935 | LC216975 | ST-8 | L | k |
| *H. cinaedi* | PAGU 614 | Hospital A, Japan | 2004 | LC216936 | LC216976 | ST-8 | L | k |
| *H. cinaedi* | PAGU 617 | Hospital A, Japan | 2004 | LC216937 | LC216977 | ST-5 | A | a |
| *H. cinaedi* | PAGU 627 | Hospital A, Japan | 2005 | LC216938 | LC216978 | ST-5 | A | a |
| *H. cinaedi* | PAGU 1024 | Hospital A, Japan | 2008 | LC216940 | LC216980 | ST-4 | B | b |
| *H. cinaedi* | PAGU 1123 | Hospital A, Japan | 2008 | LC216941 | LC216981 | ST-4 | B | b |
| *H. cinaedi* | PAGU 1124 | Hospital A, Japan | 2008 | LC216942 | LC216982 | ST-4 | B | b |
| *H. cinaedi* | PAGU 1125 | Hospital A, Japan | 2008 | LC216943 | LC216983 | ST-4 | B | b |
| *H. cinaedi* | PAGU 1411 | Hospital A, Japan | 2009 | LC216951 | LC216991 | ST-4 | C | c |
| *H. cinaedi* | PAGU 1459 | Hospital A, Japan | 2009 | LC216952 | LC216992 | ST-4 | C | c |
| *H. cinaedi* | PAGU 1496 | Hospital A, Japan | 2010 | LC216953 | LC216993 | ST-8 | M | k |
| *H. cinaedi* | PAGU 1500 | Hospital A, Japan | 2010 | LC216954 | LC216994 | ST-4 | C | c |
| *H. cinaedi* | PAGU 1513 | Hospital A, Japan | 2010 | LC216955 | LC216995 | ST-4 | C | c |
| *H. cinaedi* | PAGU 1625 | Hospital A, Japan | 2011 | LC216956 | LC216998 | ST-3 | H | h |
| *H. cinaedi* | PAGU 1679 | Hospital A, Japan | 2011 | LC216959 | LC216999 | ST-3 | H | h |
| *H. cinaedi* | PAGU 1703 | Hospital A, Japan | 2011 | LC216960 | LC217000 | ST-16 | P | n |
| *H. cinaedi* | PAGU 1708 | Hospital A, Japan | 2011 | LC216961 | LC217001 | ST-16 | P | n |
| *H. cinaedi* | PAGU 1725 | Hospital A, Japan | 2012 | LC216962 | LC217002 | ST-16 | Q | n |
| *H. cinaedi* | PAGU 1734 | Hospital A, Japan | 2012 | LC216963 | LC217003 | ST-16 | Q | n |
| *H. cinaedi* | PAGU 1735 | Hospital A, Japan | 2012 | LC216964 | LC217004 | ST-16 | Q | n |
| *H. cinaedi* | PAGU 1930 | Hospital A, Japan | 2014 | LC216972 | LC217012 | ST-18 | O | l |
| *H. cinaedi* | PAGU 1931 | Hospital A, Japan | 2014 | LC216973 | LC217013 | ST-18 | O | l |
| *H. cinaedi* | PAGU 1932 | Hospital A, Japan | 2014 | LC216974 | LC217014 | ST-18 | O | l |
| *H. cinaedi* | PAGU 1279 | Hospital B, Japan | 2008 | LC216944 | LC216984 | ST-10 | N | m |
| *H. cinaedi* | PAGU 1281 | Hospital B, Japan | 2008 | LC216945 | LC216985 | ST-10 | N | m |
| *H. cinaedi* | PAGU 1283 | Hospital B, Japan | 2008 | LC216946 | LC216986 | ST-10 | N | m |
| *H. cinaedi* | PAGU 1285 | Hospital B, Japan | 2008 | LC216947 | LC216987 | ST-11 | N | m |
| *H. cinaedi* | PAGU 1286 | Hospital B, Japan | 2008 | LC216948 | LC216988 | ST-11 | N | m |
| *H. cinaedi* | PAGU 1287 | Hospital B, Japan | 2008 | LC216949 | LC216989 | ST-11 | N | m |
| *H. cinaedi* | PAGU 1294 | Hospital B, Japan | 2008 | LC216950 | LC216990 | ST-3 | I | h |
| *H. cinaedi* | PAGU 1632 | Hospital C, Japan | 2011 | LC216957 | LC216996 | ST-9 | N | m |
| *H. cinaedi* | PAGU 1633 | Hospital C, Japan | 2011 | LC216958 | LC216997 | ST-9 | N | m |
| *H. cinaedi* | PAGU 1811 | Hospital D, Japan | 2013 | LC216969 | LC217009 | ST-16 | R | n |
| *H. cinaedi* | PAGU 1919 | Hospital E, Japan | 2014 | LC216970 | LC217010 | ST-16 | S | o |
| *H. cinaedi* | PAGU 1922 | Hospital F, Japan | 2014 | LC216971 | LC217011 | - | J | i |
