# Supplementary material for: Diversity and microevolution of CRISPR loci in Helicobacter cinaedi
Source: PLoS One. 2017 Oct 13;12(10):e0186241. doi: 10.1371/journal.pone.0186241 (PMC5640232; doi:10.1371/journal.pone.0186241)
Supplement: S2 Table — (DOCX) [file pone.0186241.s002.docx]

S2 Table. Primers designed for the CRISPR sequence amplification of *Helicobacter cinaedi.*

| Primer names | Primer sequences (5'-3') | Target strain (spacer) |
| --- | --- | --- |
| **CRISPR PCR set** |  |  |
| CRISPR1_Forward | CAATTTAGAAAACGCAGAGCC | All *H. cinaedi* strains |
| CRISPR1_Reverse | GATATGATTTACCCTGCGGAAG | All *H. cinaedi* strains |
| CRISPR2_Forward | TGTCATACTGAGACTTTTGCC | All *H. cinaedi* strains |
| CRISPR2_Reverse | GCTACCCAAAGTCGCCAAAAC | All *H. cinaedi* strains |
| **CRISPR sequencing primer** |  |  |
| CRISPR1 genotype1 seq_F | GTCTCTGCTGTTCACTATTTTTAG | genotype 1 (spacer 1I) |
| CRISPR1 genotype1 seq_R | CGCCACGCTGTAATCGTTATCTGC | genotype 1 (spacer 2P) |
| CRISPR1 genotype1 seq_R2 | TTCTGCTATCTGACACCATAGCGGTC | PAGU1752 (spacer 2Q) |
| CRISPR1 genotype1 seq_F2 | TATTGGAGCAATCGCATTAAAACC | PAGU1749 (spacer 1J) |
| CRISPR1 genotype2 seq_F | GGATTTATTTGAGTCTCTTGCTCC | genotype 2 (spacer 3J) |
| CRISPR1 genotype2 seq_R | GCAAAACAACATTAACTCTTTGATC | genotype 2 (spacer 5B) |
| CRISPR1 genotype2 seq_F2 | GATTTTGAATAGACTGTTGAACAAG | genotype 2 (spacer 3V) |
| CRISPR1 genotype2 seq_R2 | CCAAAGTTGTGTTGTATCAACC | genotype 2 (spacer 4Q) |
| CRISPR1 genotype6 seq_F | TAACCCAATGGTGAATTTGG | genotype 6 (spacer 8G) |
| CRISPR1 genotype6 seq_R | CGGAATTAAGATTCATACGCCG | genotype 6 (spacer 9F) |
| CRISPR1 genotype6 seq_R2 | CAAGTATATTGTTTGCCTC | genotype 6 (spacer 8T) |
| CRISPR1 genotype6 seq_R3 | GTTGGAGTCCCTTGACTATG | PAGU1919 (spacer 8N) |
