# Supplementary figures and images for: Diversity and microevolution of CRISPR loci in Helicobacter cinaedi
Source: PLoS One. 2017 Oct 13;12(10):e0186241. doi: 10.1371/journal.pone.0186241 (PMC5640232; doi:10.1371/journal.pone.0186241)

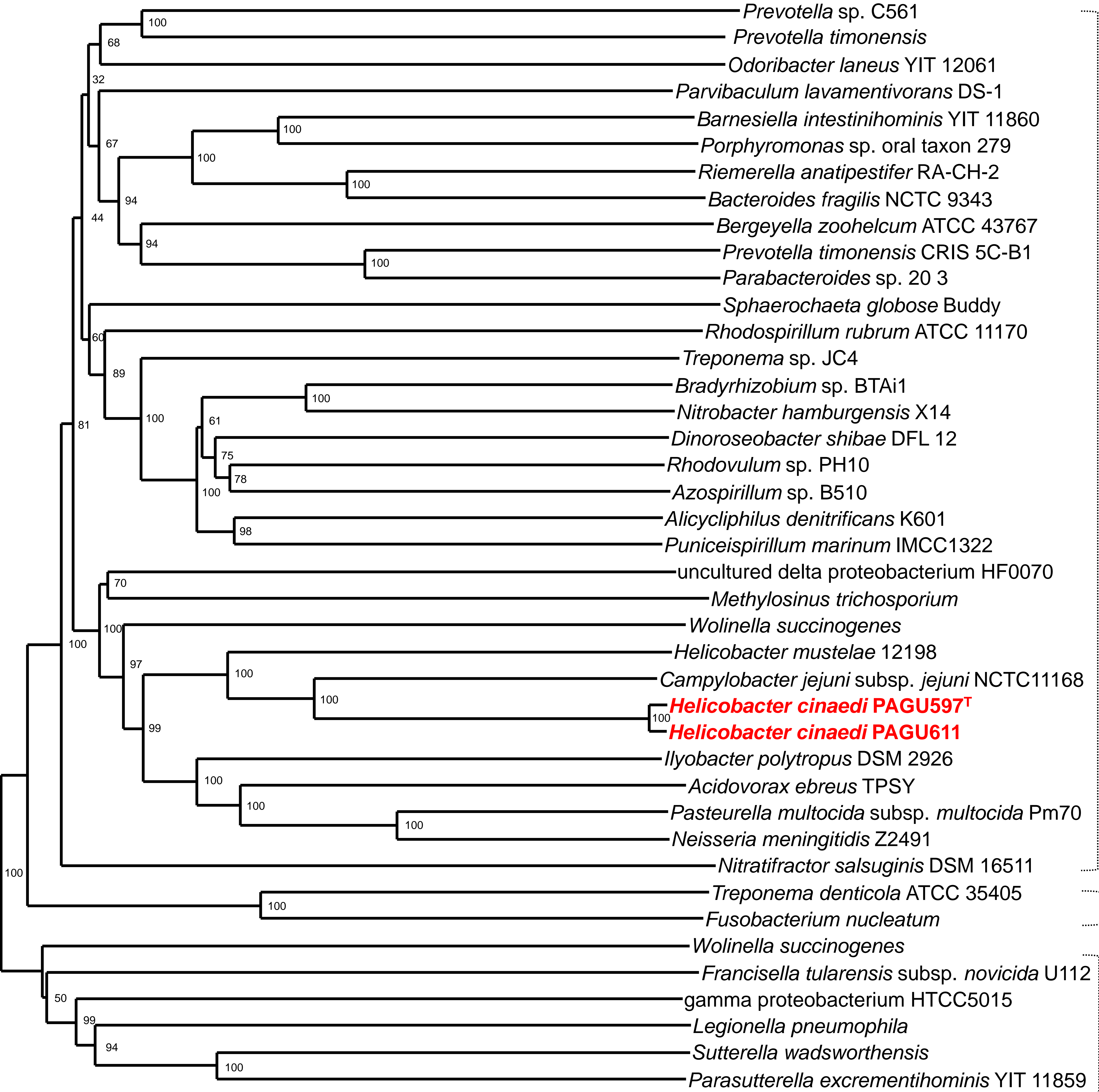

II-C

II-A

II-B

0.05

Supplement: S1 Fig — Cas9 proteins from Gram-negative type II system-containing bacteria are referenced [17]. A phylogenetic tree based on Cas9 proteins was constructed by the neighbor-joining method. H. cinaedi strains PAGU597T and PAGU611 are shown in red. Two Cas9 protein sequences were obtained from the DDBJ (Accession Nos. AP012492 and AP012344, respectively). (PDF) [file pone.0186241.s003.pdf]

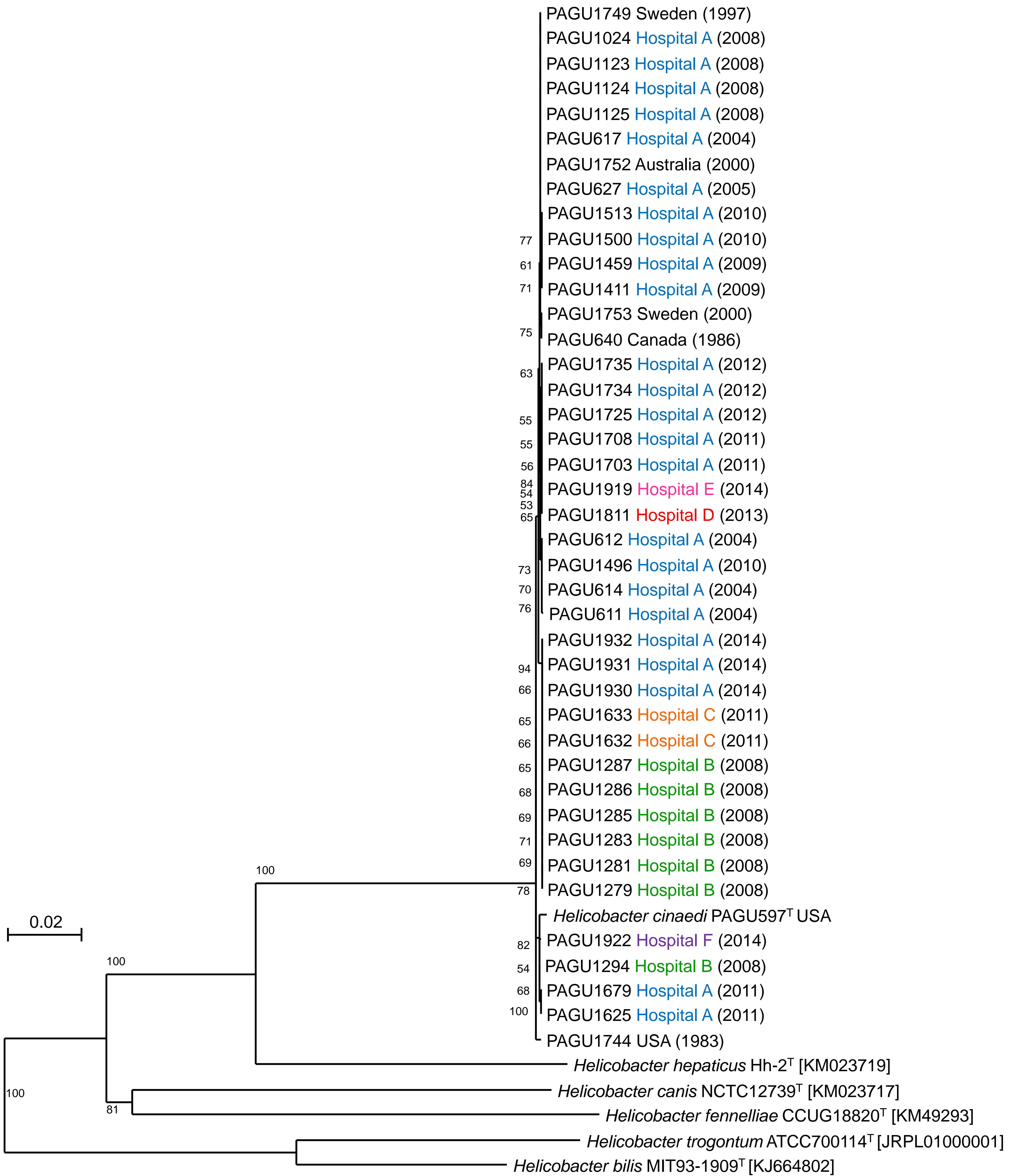

Supplement: S2 Fig — Phylogenetic analysis of the gyrA gene was performed using the neighbor-joining method. All sequences are labeled by strain number, hospital, and year of isolation. The colors represent the different hospitals. Bars: 0.02 substitutions per nucleotide position. (PDF) [file pone.0186241.s004.pdf]

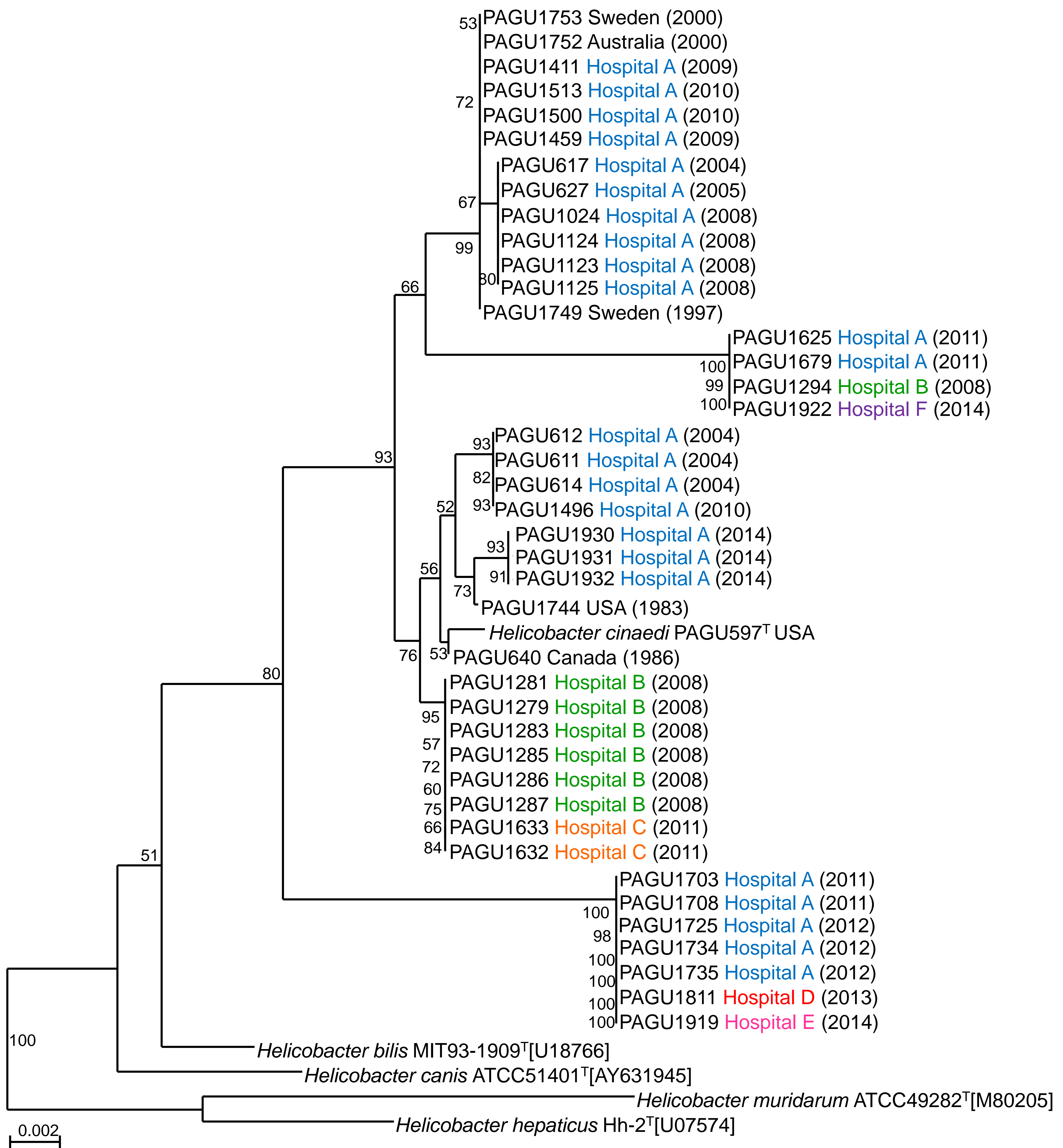

Supplement: S3 Fig — Phylogenetic analysis of the 16S rRNA gene was performed using the neighbor-joining method. Numbers at the nodes represent bootstrap values > 50% (obtained from 100 resamplings). All sequences are labeled by strain number, hospital, and year of isolation. The colors represent the different hospitals. Bars: 0.002 substitutions per nucleotide position. (PDF) [file pone.0186241.s005.pdf]
